# Single-shot bevacizumab for cerebral radiation injury

**DOI:** 10.1186/s12883-021-02103-0

**Published:** 2021-02-17

**Authors:** Martin Voss, Katharina J. Wenger, Emmanouil Fokas, Marie-Thérèse Forster, Joachim P. Steinbach, Michael W. Ronellenfitsch

**Affiliations:** 1 Dr. Senckenberg Institute of Neurooncology, University Hospital Frankfurt, Goethe University, Frankfurt/Main, Germany; 2 University Cancer Center Frankfurt (UCT), University Hospital Frankfurt, Goethe University, Frankfurt/Main, Germany; 3 German Cancer Consortium (DKTK), Partner Site Frankfurt/Mainz, Frankfurt/Main, Germany; 4 Frankfurt Cancer Institute (FCI), Georg-Speyer-Haus, Frankfurt/Main, Germany; 5 Institute of Neuroradiology, University Hospital Frankfurt, Goethe University, Frankfurt/Main, Germany; 6 Department of Radiotherapy and Oncology, University Hospital Frankfurt, Goethe University, Frankfurt/Main, Germany; 7 Department of Neurosurgery, University Hospital Frankfurt, Goethe University, Frankfurt/Main, Germany

**Keywords:** Radiation necrosis, Bevacizumab, Dexamethasone, Side effect, Edema

## Abstract

**Background:**

Cerebral radiation injury, including subacute radiation reactions and later stage radiation necrosis, is a severe side effect of brain tumor radiotherapy. A protocol of four infusions of the monoclonal antibody bevacizumab has been shown to be a highly effective treatment. However, bevacizumab is costly and can cause severe complications including thrombosis, bleeding and gastrointestinal perforations.

**Methods:**

We performed a retrospective analysis of patients treated in our clinic for cerebral radiation injury who received only a singular treatment with bevacizumab. Single-shot was defined as a singular administration of bevacizumab without a second administration during an interval of at least 6 weeks.

**Results:**

We identified 11 patients who had received a singular administration of bevacizumab to treat cerebral radiation injury. Prior radiation had been administered to treat gliomas (ten patients) or breast cancer brain metastases (one patient). 9 of 10 patients with available MRIs showed a marked reduction of edema at first follow-up. Discontinuation of Dexamethasone was possible in 6 patients and a significant dose reduction could be achieved in all other patients. One patient developed pulmonary artery embolism 2 months after bevacizumab administration. The median time to treatment failure of any cause was 3 months.

**Conclusions:**

Single-shot bevacizumab therefore has meaningful activity in cerebral radiation injury, but durable control is rarely achieved. In patients where a complete protocol of four infusions with bevacizumab is not feasible due to medical contraindications or lack of reimbursement, single-shot bevacizumab treatment may be considered.

## Background

Radiation necrosis has been reported in approximately 6 % of patients with brain tumors after radiation therapy and can lead to significant morbidity and, if untreated, mortality by progressive necrosis and brain edema [1]. Additionally, the risk of misinterpreting radiation injury (including subacute radiation reactions and later stage radiation necrosis) for tumor progression can prevent adequate therapy [2]. The risk of radiation injury is highest in patients who undergo repeated courses of radiotherapy, even with prolonged intervals between the two treatments. Lee et al. reported a rate of 64 % radiation necrosis for hypofractionated re-irradiation (45 Gy in 15 fractions) in glioma patients at least 12 months post-treatment [3]. Conceptually, radiation-induced injury is thought to result from damage to vascular endothelial and glial cells. Secretion of vascular endothelial growth factor (VEGF)-A appears to be responsible for edema formation via increasing vascular permeability and inducing a pro-inflammatory environment [4].

Bevacizumab is an antibody targeting VEGF-A induced angiogenesis and has been evaluated as a treatment for malignant brain tumors. While several phase III trials of first-line therapy failed to show any effect on overall survival [5–7], there was still a pronounced effect of bevacizumab on the blood brain barrier with reduced gadolinium contrast enhancement and edema reducing the rate of pseudoprogression in MRI scans from 9.3 to 2.2 % in the AVAglio trial [8]. Bevacizumab has also been used in small clinical trials as a treatment for radiation necrosis. Levin et al. reported a randomized, placebo-controlled trial of four infusions of bevacizumab 7.5 mg/kg at 3-week intervals for radiation necrosis of the central nervous system [9]. This trial demonstrated an impressive clinical and radiological improvement in all patients receiving bevacizumab while no patient with placebo treatment improved spontaneously. This treatment efficacy came at the cost of a high rate of adverse events in the bevacizumab group (6 of 11 patients) while no adverse events occurred in the placebo group. Common serious side effects of bevacizumab regimens include pulmonary artery embolism, venous thrombosis and intracranial hemorrhage [10, 11]. Whether a reduced number of bevacizumab cycles could also suffice to adequately treat radiation injury with a potentially reduced side effect profile remains unclear. In tumor treatment, clinical trials comparing standard and low-dose bevacizumab regimens found no significant differences in efficacy [12] but suggested a more favorable toxicity profile [13, 14].

Bevacizumab has not been approved by the European Medicines Agency (EMA), neither for progressive glioblastoma nor for the treatment of radiation reaction (FDA approval for adult patients with progressive glioblastoma) but is often used as an individual, off-label therapy for dexamethasone-refractory radiation necrosis or following steroid discontinuation due to adverse effects. In cases of rapid clinical deterioration, immediate treatment with bevacizumab can be considered to prevent permanent damage to eloquent areas. However, reimbursement by insurance companies can be difficult. If a singular administration (single-shot) of bevacizumab, that is considered financially affordable, was sufficient to treat cerebral radiation injury, this would broaden options for both, patients and physicians due to lower financial as well as potential side effect risks, especially for patients with prior vascular contraindications.

## Methods

We performed a retrospective analysis of patients treated in our clinic between 2016 and 2019 to identify patients with cerebral radiation side effects who received a singular treatment with bevacizumab. Diagnosis of acute radiation reaction and radiation necrosis had been made in the interdisciplinary tumor board based on MRI and considering the field of radiation and the time from last radiation therapy (results section). Single-shot bevacizumab was defined as a singular administration of bevacizumab without a second administration during an interval of at least six weeks. The patient collective was evaluated with regard to histology, patient age at diagnosis of radiation injury, duration and maximum dose of dexamethasone, clinical course and possible side-effects, as well as the radiologic response to bevacizumab treatment. MRI scans included at least axial fluid-attenuated inversion recovery (FLAIR), T2-weighted, and T1-weighted images before and after application of gadolinium-based contrast agent. The extent of edema was estimated on the axial FLAIR or T2-weighted sequence. Response to bevacizumab treatment was defined as a reduction of the edema by at least 25 % [9].

Written consent by the individual patient for this retrospective data collection was waived by the ethics committee of the University Hospital Frankfurt; Goethe University which also approved the access to the patients’ data (IRB decision # 4/09, project SNO_01–08). Microsoft Excel was used for data management and analysis. Corel Draw 2019 was used to create figures.

## Results

From 2016 until the end of 2019 approximately 400 patients received radiation of the brain for any reason (brain tumor or brain metastasis including primary therapy and re-irradiation therapy) at our cancer center. During this time, about 65 patients were treated with bevacizumab for radiation reaction. Retrospective analysis revealed 11 patients who were initially treated with a single-shot (Table 1). Ten patients had received prior fractionated radiation therapy for gliomas including 2 patients being treated primarily by radiotherapy at initial diagnosis (radiotherapy doses: 54 and 60 Gy) and 8 patients, who underwent re-irradiation for recurrent tumor (radiotherapy doses: 20–36 Gy), whereas one patient received re-irradiation (dose: 30 Gy) for recurrent brain metastasis of breast cancer after an initial radiosurgery.

**Table 1:** Patient characteristics

**Number of Patients**	11
**Age at treatment with BEV [years]**	
Median (range)	47 (22 – 73)
**Histology**	
Glioma	91% (10)
Brain metastasis (breast cancer)	9% (1)
**Radiation for**	
Recurrent tumor	82% (9)
Primary therapy	18% (2)
**Last radiation therapy prior to BEV [Gy]**	
5x4	18% (2)
10x3	9% (1)
10x3,5	36% (4)
12x3	9% (1)
15x2,67	9% (1)
30x1,8	9% (1)
30x2	9% (1)
**Time from radiation to diagnosis of radiation injury [months]**	
Median (range)	2 (1-7)
**Maximum Dose of dexamethasone [mg]**	
Before therapy, Median (range) After therapy, Median (range)	8 (0 – 40) 0 (0 - 4)
**Karnofsky-Score [%]**	
Before therapy, Median (range)	50 (40 – 80)
After therapy, Median (range)	60 (40 – 80)
**Dose of BEV single-shot**	
7,5mg/kg	73% (8)
10mg/kg	27% (3)
**Reported benefit by patient**	
Yes	64% (7)
No	36% (4)

Abbreviation: *BEV* bevacizumab

As soon as acute radiation reaction / radiation necrosis was diagnosed, therapy with dexamethasone was started or an already established therapy with steroids was intensified following a mean interval of 2 months post-radiation therapy. Median peak dose of dexamethasone was 8 mg/day, with a maximum dose of 40 mg/day in 2 patients. Diagnosis of radiation injury was based on MRI in 10 patients using additional MR-perfusion in 6 patients. In one patient, diagnosis was confirmed by positron emission tomography (F-18-fluroethyltyrosine). In no case had a biopsy been performed to confirm the diagnosis histologically.

When dexamethasone did not improve clinical symptoms or could not be tolerated at the required doses due to side effects, off-label treatment with bevacizumab was recommended at the institutional multidisciplinary tumor board. Four of these patients had a single-shot of bevacizumab treatment because of a high-risk situation for side effects rendering long-term repeat treatment with bevacizumab unfeasible (pulmonary embolism, deep vein thrombosis, fracture of several rips, hemorrhage of the tumor). In another patient there were concerns of possible increased toxicity as the patient received ongoing therapy with lomustin and temozolomide [15], and in a further patient bevacizumab was only administered once because of the ensuing palliative setting aimed at improving aphasia (Patient 1). Moreover, one patient initially received one singular infusion due to personal concerns with regard to side effects (patient 11), and two did not consent to further infusions (Patient 6 and patient 9). Two patients did not receive reimbursement by the insurance company for further treatment after the single-shot of bevacizumab.

Eight patients received 7.5 mg/kg as proposed by Levin et al., three patients received 10 mg/kg as used in the neuro-oncological trials for bevacizumab at that time [6, 9]. The treatment was well-tolerated without any acute side effects during the infusion. One patient with immobility developed deep vein thrombosis with subsequent pulmonary artery embolism two months after bevacizumab.

**Fig. 1:**
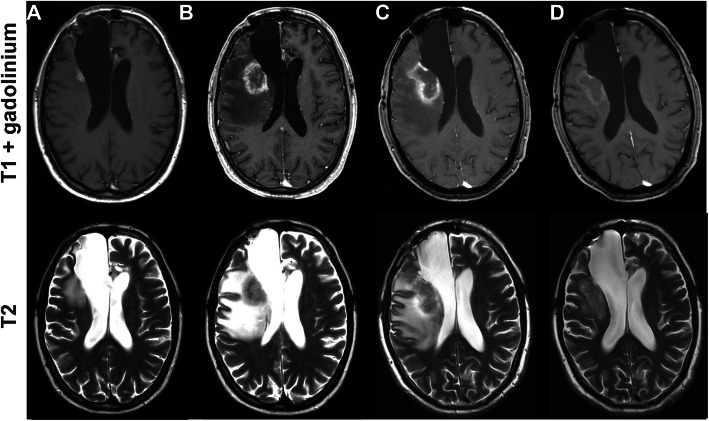
MRI scans of a 34 year old patient with IDH-mutated astrocytoma. **a** MRI revealed a small recurrent tumor adjacent to the dorsal resection cavity with small surrounding edema. **b** The patient was treated with re-radiation therapy with 35 Gy and concomitant temozolomide. First MRI after the treatment showed an increase of contrast enhancement and edema which was diagnosed as radiation necrosis. **c** Therapy with 8 mg of dexamethasone did neither improve the MRI nor the clinical symptoms and bevacizumab 7.5 mg/kg was administered as a single-shot. **d** First scan one month later displayed a marked reduction in contrast enhancement and of the edema. Treatment with dexamethasone could be stopped. The follow up 3 months later was stable (not shown)

After a median interval of 55 days following the administration of bevacizumab first MRI showed a marked reduction of brain edema (at least 25 %) in 9/10 evaluable patients. An example is given in Fig. 1.

After single-shot bevacizumab, patients Karnofsky Performance Score (KPS) improved from a median of 50–60 % and 7 patients reported markedly improved clinical symptoms at the first visit after bevacizumab. Here, we noticed that the only slight improvement of KPS underestimated the clinical benefit in the activity of daily life. Indeed, the ability for an independent transfer from the wheelchair to a bed or toilet has a great impact on the patient’s quality of life that is not accurately reflected in the Karnofsky-Index. Notably, dexamethasone could be stopped in 6 of the patients. In all other patients, the dose of dexamethasone could be gradually reduced, finally reaching doses between 0.5 and 4 mg/day after a median time of 39 days after the single-shot.

**Fig. 2:**
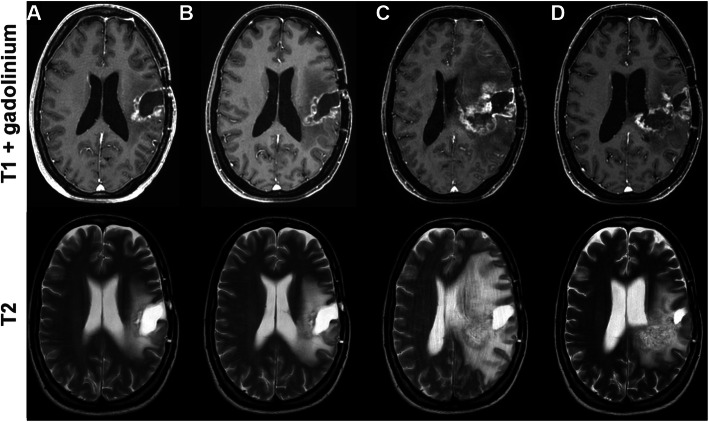
MRI scans of a 33 year old patient with IDH-wildtype glioblastoma. First (**a**) and second (**b**) MRI after resection and radiation therapy of recurrent glioblastoma showed not signs of tumor progression. Dexamethasone was started because of clinical deterioration before the third control (**c**) which showed a substantial increase in contrast enhancement and edema which were interpreted as late radiation necrosis. Bevacizumab 7.5 mg/kg was administered as a single-shot. **d** First scan 1.5 months later displayed a marked reduction of the edema while there was only a minor reduction of contrast enhancement. Diagnosis was changed from radiation necrosis to recurrent tumor

**Fig. 3:**
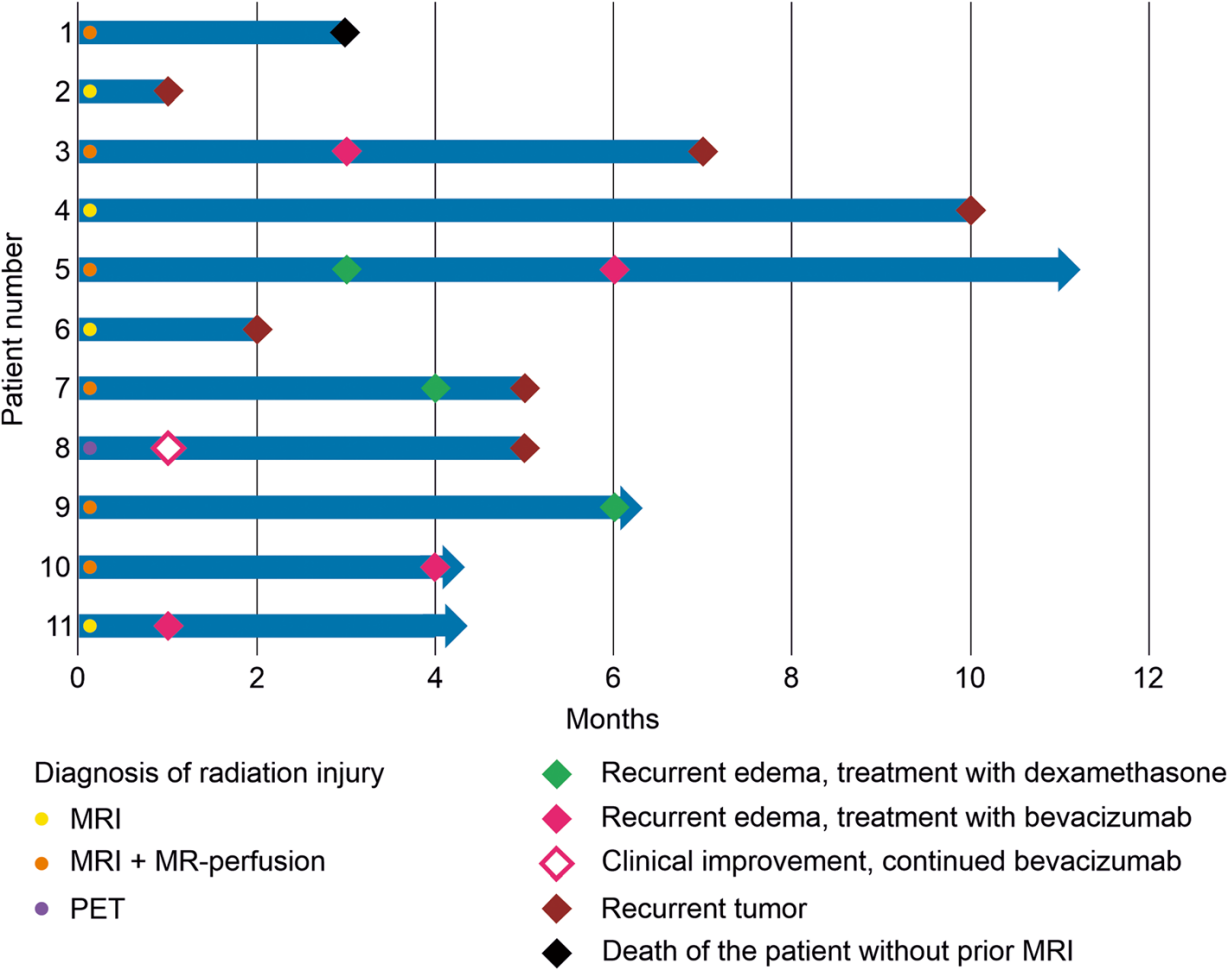
Time to treatment failure. The swimmer plot shows the course of the individual patients labeled at the left side. The radiological diagnosis is indicated by color-coded dots (yellow: MRI, orange: MRI and MR-perfusion, purple: PET). The color-coded diamonds indicate the treatment failure of the single-shot bevacizumab (green: treatment of recurrent edema with corticosteroids, blue: treatment of recurrent edema with bevacizumab, blue border: resumed bevacizumab as the symptoms did not completely resolve, red: recurrent tumor, black: death of the patient because of recurrent tumor). Median time from single-shot to time of treatment failure of any cause) was three months

Mean time to treatment failure was 3 months (range 1–10 months). Importantly, treatment failure to bevacizumab was due to tumor progression (patient 2, 4 and 6) or death (patient 1) in four patients, therefore, tumor progression should always be taken into account when interpreting clinical deterioration as the latter likely reflects a mixture of tumor progression and radiation necrosis (Fig. 2). One patient (patient 8) had both a marked improvement in clinical symptoms and MRI with a decline in contrast enhancement after single-shot. In this patient, however, treatment with bevacizumab was resumed 8 weeks after the first infusion, since the patient still experienced disabilities in the activities of daily life, and the single-shot had been tolerated well. Treatment failure in the other patients was diagnosed due to recurrent edema in follow-up MRI with or without clinical symptoms (Fig. 3).

## Discussion

Cerebral irradiation is an integral part of the treatment of brain cancer. One of the most severe complications is cerebral necrosis that can occur in patients with primary or metastatic brain tumors especially after a second course of irradiation for recurrent tumors. Despite promising efficacy in the treatment of cerebral radiation necrosis from smaller clinical trials, no application for bevacizumab approval for this indication has been filed. Reimbursement by insurance companies therefore remains difficult and is granted only on a case by case basis limiting the availability of bevacizumab. Assessing the efficacy of a singular bevacizumab treatment with a potentially more favorable side-effect profile and lesser financial burden than the cyclic treatment addresses a clinically important challenge. In the present work we show that a singular dose of bevacizumab resulted in significantly reduced edema on MRI sequences in all evaluable patients with two-thirds of patients reporting a meaningful improvement of clinical symptoms. In this context the very shot interval from radiation therapy to the development of brain lesions has to be noted. The mean time of two month is rather short for radiation necrosis in comparison to the trial by Levin et al. [9]. Therefore it is plausible that some of the patients suffered from a subacute or early delayed radiation reaction rather than manifest necrosis. Despite the small series, this study provides encouraging data, indicating that singular administration of bevacizumab might be a useful option for the treatment of radiation reaction / necrosis, especially in patients where prolonged bevacizumab treatment is not deemed feasible, as for example due to prior thromboembolic events or due to denial of therapy reimbursement. Additionally, even when bevacizumab is available for multiple treatments, a single-shot might be sufficient treatment for some patients. At the present time it is unclear whether a resumption of therapy in cases where a single-shot is not sufficient has disadvantages on the course of cerebral radiation necrosis.

In our analysis, we identified only one potentially severe side effect in one patient who was diagnosed with pulmonary artery embolism two months after bevacizumab. Whether this was instead attributable to immobility of the patient, who was later also diagnosed with deep vein thrombosis, or if this only contributed to the embolism remains unclear.

An interesting variant of the single-shot bevacizumab concept to further reduce the systemic side effects could be a local administration. The ongoing LIBERTI trial (NCT02819479) evaluates the efficacy of a single, intra-arterial dose of only 2.5 mg/kg bevacizumab [16]. Despite the large molecular mass of bevacizumab some penetration of the disrupted blood-brain barrier in regions of radiation injury appears possible [17]. The reduced side effect might come at the cost of a decreased duration of edema control. With a half-life of three weeks, the effect on the blood brain barrier might not be lasting, and the downside could be a rebound phenomenon with the need of bevacizumab re-challenge, as also indicated by the short time to treatment failure in our collective of only three months. The trial of Levin et al. with four administrations of 7.5 mg/ reported a relapse of radiation necrosis in 25 % of patients [9]. Zhuang et al. reported 14 patients with cerebral radiation necrosis who were treated with a lower dose of 5 mg/kg bevacizumab for at least 3 cycles of therapy [18]. MRI showed improvement in 13 of the 14 patients, but 10 of the 13 responsive patients exhibited a rebound phenomenon in the later follow-up. One option would be to further lower the dose of bevacizumab but keep the continuous administration. This approach of lowering the dose to 1 mg/kg bevacizumab every three weeks has revealed promising results in a phase 2 trial [19]. The trial included 21 patients and the grade of the edema index was improved in 19 patients. In contrast to Levin et al., no adverse events above grade 2 were reported. This concept has been further supported by case reports with low-dose bevacizumab and even longer intervals between the administration [20].

Prophylactic administration of a singular dose bevacizumab in high-risk situations (re-irradiation therapy, large irradiation fields or/and already present widespread edema prior to irradiation) could also be an option to consider. Thereby clinical deterioration might be prevented and the need for corticosteroids as well as the risk of a rebound effect after termination of bevacizumab treatment might be reduced. Such an approach has been explored in a phase 1 trial by Clarke et al. This trial included bevacizumab treatment to intensify the radiation dose of hypofractionated stereotactic re-irradiation [21]. This concept could be even more beneficial in cyber knife radiosurgery [22, 23].

While histological confirmation of cerebral radiation necrosis is clearly limited to only the most ambiguous cases, we here report a cohort of 11 patients whose radiological scans and dynamics indicated radiation necrosis. Two patients (patient 2 and 6) had treatment failure shortly after the single-shot due to tumor progress in the MRI and these were also patients where diagnosis was based on conventional MRI without MR-perfusion or MR-spectroscopy. A more selected collective of histologically diagnosed radiation necrosis could have shown more sustained effects of bevacizumab.

## Conclusions

In summary, bevacizumab is an effective treatment for patients with cerebral radiation injury. Optimal dosing and intervals still have to be defined but most likely lower doses and longer intervals than investigated in previous trials [9] are sufficient. In the case of patients at high risk for side effects, single-shot of bevacizumab may be used as a test-dose and treatment can be continued when necessary.

## Data Availability

Raw data were generated at the Dr. Senckenberg Institute of Neurooncology. The datasets generated are available from the corresponding author on reasonable request.
